# STAT2 Limits Host Species Specificity of Human Metapneumovirus

**DOI:** 10.3390/v12070724

**Published:** 2020-07-04

**Authors:** Meredith C. Rogers, Margot Miranda-Katz, Yu Zhang, Tim D. Oury, Melissa B. Uccellini, Adolfo García-Sastre, John V. Williams

**Affiliations:** 1 Department of Pediatrics, University of Pittsburgh School of Medicine and UPMC Children’s Hospital of Pittsburgh, Pittsburgh, PA 15224, USA; meredith.rogers@vanderbilt.edu (M.C.R.); mirandakatz@wisc.edu (M.M.-K.); yuz113@pitt.edu (Y.Z.); 2 Department of Pathology, Microbiology, and Immunology, Vanderbilt University Medical Center, Nashville, TN 37212, USA; 3 Department of Pathology, University of Pittsburgh School of Medicine, Pittsburgh, PA 15261, USA; tdoury@pitt.edu; 4 Department of Microbiology, Icahn School of Medicine at Mount Sinai, New York, NY 10029, USA; Melissa.Uccellini@mssm.edu (M.B.U.); Adolfo.Garcia-Sastre@mssm.edu (A.G.-S.); 5 Global Health and Emerging Pathogens Institute, Icahn School of Medicine at Mount Sinai, New York, NY 10029, USA; 6 Department of Medicine, Division of Infectious Diseases, Icahn School of Medicine at Mount Sinai, New York, NY 10029, USA; 7 The Tisch Cancer Institute, Icahn School of Medicine at Mount Sinai, New York, NY 10029, USA; 8 Institute for Infection, Inflammation, and Immunity in Children (i4Kids), University of Pittsburgh, Pittsburgh, PA 15224, USA

**Keywords:** human metapneumovirus, STAT2, STAT1, virus tropism, innate immunity, type I interferon, acute respiratory infection

## Abstract

The host tropism of viral infection is determined by a variety of factors, from cell surface receptors to innate immune signaling. Many viruses encode proteins that interfere with host innate immune recognition in order to promote infection. STAT2 is divergent between species and therefore has a role in species restriction of some viruses. To understand the role of STAT2 in human metapneumovirus (HMPV) infection of human and murine tissues, we first infected STAT2^−/−^ mice and found that HMPV could be serially passaged in STAT2^−/−^, but not WT, mice. We then used in vitro methods to show that HMPV inhibits expression of both STAT1 and STAT2 in human and primate cells, but not in mouse cells. Transfection of the murine form of STAT2 into STAT2-deficient human cells conferred resistance to STAT2 inhibition. Finally, we sought to understand the in vivo role of STAT2 by infecting hSTAT2 knock-in mice with HMPV, and found that mice had increased weight loss, inhibition of type I interferon signaling, and a Th2-polarized cytokine profile compared to WT mice. These results indicate that STAT2 is a target of HMPV in human infection, while the murine version of STAT2 restricts tropism of HMPV for murine cells and tissue.

## 1. Introduction

Human metapneumovirus (HMPV) is a negative sense single-stranded RNA virus and a member of the *Pneumoviridae* family with its closest related human pathogen respiratory syncytial virus (RSV) [1]. HMPV is a leading cause of severe respiratory illness in children and adults [2,3,4,5,6], and causes significant morbidity and mortality in immunocompromised hosts [4,7,8,9]. Even though virtually all humans are infected with HMPV by the age of 5 years [10,11], reinfection with HMPV is common, which can lead to particularly poor outcomes in immunocompromised and elderly patients [2,3,4,7,8,9]. Due to the wide prevalence and burden of severe disease in multiple risk groups, it is essential to better understand the virus and host factors that are involved in promoting and restricting HMPV infection.

Many viruses encode proteins that interfere with interferon (IFN) signaling or inhibition of IFN stimulated genes (ISGs) [12]. Pneumoviruses and the related paramyxoviruses antagonize host IFN signaling, specifically STAT1 (signal transduction and activator of transcription) and/or STAT2. Paramyxoviruses utilize alternate transcripts of the phosphoprotein [13], while RSV encodes the nonstructural proteins NS1 and NS2 that suppress STAT1/STAT2 signaling [14]. Despite being related to RSV and paramyxoviruses, none of the nine proteins encoded by the HMPV genome have homology with known pneumovirus or paramyxovirus inhibitors of STAT1 or STAT2 [15,16]. In spite of this, HMPV was shown to inhibit phosphorylation of STAT1 in cell lines and primary human epithelial cells [17]. Recent work in our lab identified the HMPV small hydrophobic (SH) protein as necessary and sufficient to inhibit phosphorylation of STAT1 [18]. Others found no inhibition of STAT2 by HMPV; however, these experiments used a relatively low multiplicity of infection (MOI) in cell culture [17,19]. Other groups have also shown roles for SH [20,21] as well as the HMPV proteins G and M2-2 [22,23,24] in the inhibition of innate immunity.

Viruses have tropism for cell types, tissues, and host species, which explains why natural infections only cause certain symptoms in certain species. Tropism can be caused by a host cell lacking a viral entry receptor, or by the virus’ ability to circumvent host immunity only in certain cells or species. Mouse models for HMPV have been developed, but mice are only semi-permissive for HMPV, requiring a high viral inoculum for productive in vivo infection ([25] and unpublished observations), which may be due to species differences in the innate immune proteins antagonized by the virus. For example, human and mouse STAT1 have ~95% amino acid similarity, while human and mouse STAT2 are relatively divergent, with only ~70% similarity [26,27]. As a consequence of this sequence divergence, murine STAT2 restricts the related human viruses RSV, hPIV2, and hPIV5 in cell culture [28,29]. We sought to test the hypothesis that HMPV may similarly be restricted by STAT2 in a species-specific manner.

In this study, we used in vitro and in vivo approaches to determine the effect of HMPV on human and murine STAT1/2. We found that HMPV antagonized expression and nuclear localization of both STAT1 and STAT2 in primate cells but not murine cells. Transfection of STAT2-deficient U6A cells with hSTAT2 or mSTAT2 revealed that suppression of both hSTAT1 and hSTAT2 were prevented by mSTAT2. In vivo, HMPV infection of hSTAT2 knock-in (hSTAT2 KI) mice led to greater weight loss, inhibition of interferon stimulated genes, and a Th2-skewed cytokine profile compared to WT mice. These data indicate that mSTAT2 restricts HMPV’s ability to inhibit both STAT1 and STAT2 and suggest that productive infection of humans by HMPV is partly due to inhibition of hSTAT2 antiviral signaling.

## 2. Materials and Methods

### 2.1. Cells and Plasmids

The following cell lines were used: BEAS2b (ATCC CRL-9609), Vero E6 (ATCC CCL-81), NIH/3T3 (ATCC CRL-1658), U3A (ECACC) (ATCC: Manassas, VA, USA; ECACC: Salisbury, UK). CMT64/61 (ECACC) and U6A (ECACC) cells were purchased from Sigma (St. Louis, MO, USA). All cell lines were maintained, infected, and transfected in DMEM supplemented with 10% FBS. pUNO1-hSTAT2 and pUNO1-mSTAT2 plasmids were purchased from Invivogen (San Diego, CA, USA).

### 2.2. Viruses

HMPV clinical strain TN/94-49 (subtype A2) was grown in LLC-MK2 cells (ATCC CCL-7) and virus titers measured by plaque assay in LLC-MK2 cells as previously described [25].

### 2.3. In Vitro Infection

For cell experiments, cells were inoculated with HMPV strain TN/94-49 at an MOI of 1–10 in a 24-well plate. Mock-infected cells were inoculated with media or LLC-MK2 cell lysate, which had an equivalent effect on STAT1 and STAT2 protein levels and phosphorylation [30]. Then, 16–24 h post-infection, cells were treated with 1000 U/mL human IFNα (Alpha 2a) (PBL; Piscataway, NJ, USA) for human and primate cells, or 1000 U/mL murine IFNβ (PBL) for mouse cells for 30–40 min. After treatment, media were aspirated from the tissue culture dish and cells were lysed in RIPA buffer (ThermoFisher; Waltham, MA, USA) for western blotting or fixed in 4% paraformaldehyde for immunofluorescence.

### 2.4. Transfection

Transfections were performed using Lipofectamine 2000 (Life Technologies; Carlsbad, CA, USA) following the manufacturer’s protocol with some exceptions: for each well in the 24-well plates, 2.5 μL Lipofectamine 2000 was diluted into 37.5 μL Opti-MEM (not supplemented) and was mixed with 1 μg plasmid in 37.5 μL Opti-MEM for a total volume of ~75 μL. This was incubated for 15 min before being added to wells. Fifty percent of media was replaced after 6 h. Then, 16–24 h post-transfection, cells were treated with IFNα/β as above and lysed for Western blotting.

### 2.5. Western Blotting

Cells were lysed in ice-cold RIPA buffer (Thermo Scientific, Waltham, MA, USA) containing Halt protease and phosphatase inhibitors (Thermo Scientific). Samples were centrifuged at 14,000× *g* for 15 min to pellet debris, and supernatant was used for protein analysis. Total protein from cell lysate was quantified by BCA assay (Thermo Scientific) and protein was normalized between samples. A total of 11–20 ug of protein was loaded into each well for each western blot experiment. After total protein quantification, samples were normalized and more concentrated samples were diluted in RIPA buffer to achieve equal concentrations across samples. Samples were diluted in 4× LDS sample buffer (Invitrogen; Carlsbad, CA, USA) and 10× Sample Reducing Agent (Invitrogen: Carlsbad, CA, USA) and boiled for 8 min at 95 °C. Proteins were separated on a 4–12% Bis-Tris polyacrylamide gel before transfer to a PVDF membrane. Membranes were blocked in 5% BSA in Tris-buffered saline with 0.1% Tween-20 (TBS-T) or 5% nonfat dry milk in TBS-T. Primary antibodies against STAT1 (Cell Signaling Technologies (CST, Danvers, MA, USA), D1K9Y), pSTAT1 (CST, 58D6), STAT2 (CST, D9J7L), pSTAT2 (for human, CST D3P2P; for mouse (polyclonal), EMD Millipore (Burlington, MA, USA)), actin (Hrp conjugated, Abcam (Cambridge, UK), and GFP (Invitrogen, A-11122)) were used in a 1:1000 dilution (or a 1:10,000 dilution for actin) overnight with rocking at 4 °C. After TBS-T wash, HRP-conjugated secondary antibodies against rabbit or mouse were added in 5% BSA-TBS-T or 5% milk-TBST for 1 h. Blots were washed with TBS-T and put in TBS until imaging. Western blots were developed using West Femto (Thermo Scientific) and imaged on a ChemiDoc XRS+ (BioRad, Hercules, CA, USA). Band quantification was performed using Image Lab v5.2 (BioRad).

### 2.6. Immunofluorescence

After infection and IFN treatment, cell supernatant was removed from BEAS2b cells and cells were fixed with 4% paraformaldehyde, then permeabilized with 100% methanol at −20 °C. Cells were blocked with 5% goat serum and 0.3% Triton-X100 in PBS. Primary and secondary antibodies were diluted in 1% BSA and 0.3% Triton-X100 in PBS. DAPI (5 µg/mL) was added to distinguish nuclei. Antibodies used were STAT1 (CST, D1K9Y) STAT2 (CST, D9J7L), HMPV anti-Fusion protein 54G10 [31], secondary Alexa Fluor 488-conjugated anti-human and Alexa Fluor 568-conjugated anti-rabbit (Invitrogen). All fluorescent images were equally color enhanced (increased brightness and contrast) for better visibility in publication. For quantification of nuclear STAT1 or STAT2 signal, original (non-color enhanced) fluorescent images were analyzed using Fiji ImageJ [32,33,34]. Nuclei were selected from the DAPI channel, then the mean intensity was measured in the STAT1 or STAT2 channel. For quantification of cytosolic protein, the cytosol was selected from the transmitted light image and the mean intensity was measured in the STAT1 or STAT2 channel.

### 2.7. Mice

C57BL/6 mice were obtained from Jackson Laboratories (Bar Harbor, ME, USA). STAT1^−/−^ and STAT2^−/−^ mice were from Dr. David Levy (New York University; New York, NY, USA) [35] and Dr. Christian Schindler (Columbia University; New York, NY, USA) [36], courtesy of Dr. John Alcorn. hSTAT2 KI mice were from Adolfo Garcia-Sastre (Icahn School of Medicine at Mount Sinai; New York, NY, USA) [37]. Male and female 6–14-week-old mice were used for all experiments, with control groups age and sex matched. All mice were bred and maintained in specific pathogen free conditions in accordance with the Institutional Animal Care and Use Committee of University of Pittsburgh (Protocol #18032376, approved 1 March 2018). 

### 2.8. In Vivo Infection and Serial Passage

For all animal experiments, mice were anesthetized with ketamine-xylazine (mouse passaging experiments) or isofluorane (hSTAT2 KI experiments) and intranasally (for mouse passaging) or intratracheally (for hSTAT2 KI) infected with 1–5 × 10^6^ PFU/mL HMPV TN 94-49, or lung homogenate from a previously infected mouse, in a 100-µL volume. For serial passage, mice were euthanized at day 5 post-inoculation. Lungs were harvested and homogenized in 1–2 mL 0% Opti-MEM in a glass Tenbroeck homogenizer as previously described [25]. Lung homogenates were clarified by centrifugation at 1200 RPM (300× *g*) for 10 min. Clarified lung homogenate was aliquoted into cryovials and snap-frozen in an ethanol dry ice bath before storage at −80 °C. For serial passage, clarified lung homogenate was pooled from 2–3 mice before inoculation into recipient mice.

### 2.9. Histology

The left lobe of the lung was inflated and fixed in formalin, then subsequently sectioned and stained with H&E, with groups combined into a single slide. All fields of each H&E-stained slide were scanned at 200× magnification, and each field was scored as follows: 0: normal lung tissue, 1: 0% to 25% of tissue area with inflammation, 2: 25–50% of tissue area with inflammation, 3: 50–75% of tissue area with inflammation, or 4: 75–100% of tissue area with inflammation [38]. The frequency of each inflammation score was combined by group. Inflammation scores were combined as follows to allow for statistical analysis by Fisher’s exact test: scores of 0–2 were categorized as mild/moderate inflammation and scores of 3–4 were categorized as severe inflammation. 

### 2.10. Quantitative RT-PCR

Lung homogenate from the whole lung was frozen at −80 °C until use for quantitative RT-PCR (qPCR). RNA was extracted from the lung homogenate with the PureLink RNA mini kit (Thermo Fischer Scientific, Waltham, MA, USA) according to manufacturer instructions and stored at −80 °C until further use. Five microliters of extracted RNA were used for qRT-PCR in 25-µL reaction mixtures on an ABI StepOnePlus Real-Time PCR System (Thermo Fisher Scientific) using the AgPath-ID One-Step RT-PCR Kit (Thermo Fisher Scientific). TaqMan primers and probes were used according to manufacturer’s instructions (all Thermo Fisher Scientific). All values were normalized to the housekeeping gene Hprt, and fold change in chemokine was measured in infected groups compared to mock-infected controls using the ΔΔ cycle threshold method.

### 2.11. Multiplex Cytokine Analysis

Lung homogenate from the whole lung was used for cytokine analysis by Cytokine & Chemokine 36-Plex Mouse ProcartaPlex Panel 1A (Invitrogen) according to manufacturer’s instructions. The following cytokines and chemokines were measured: IFN gamma; IL-12p70; IL-13; IL-1 beta; IL-2; IL-4; IL-5; IL-6; TNF; GM-CSF; IL-18; IL-10; IL-17A; IL-22; IL-23; IL-27; IL-9; GRO alpha; IP-10; MCP-1; MCP-3; MIP-1 alpha; MIP-1 beta; MIP-2; RANTES; eotaxin; IFN alpha; IL-15/IL-15R; IL-28; IL-31; IL-1 alpha; IL-3; G-CSF; LIF; ENA-78/CXCL5; M-CSF.

### 2.12. Statistical Analysis

Data were analyzed using Prism version 8.0 (GraphPad Software; San Diego, CA, USA). A one-tailed Student’s *T* test was used to analyze fold change from Western blots. Fisher’s exact test was used to analyze differences in histology inflammation scores. A two-tailed unpaired Student’s *T* test was used to analyze comparisons between two groups. For comparisons between multiple groups, a one- or two-way ANOVA was performed. Error bars on graphs represent SD.

## 3. Results

### 3.1. HMPV Grows to Significantly Higher Titer in STAT2^−/−^ Mice

**Figure 1:**
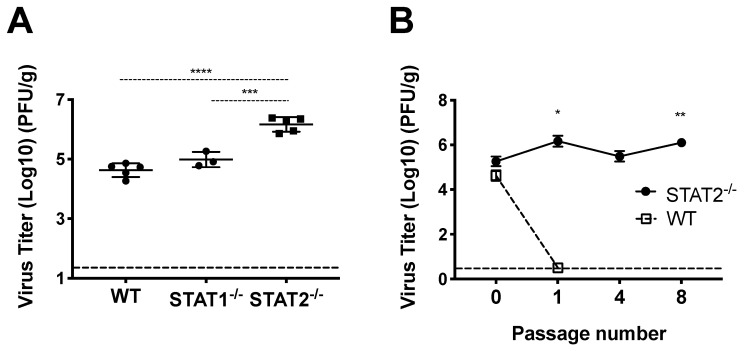
HMPV infection of and STAT2^−/−^ mice. (**A**) Mice were infected with HMPV TN/94-49 and euthanized at day five of infection. Lung virus titers were determined by plaque assay. (**B**) HMPV TN/94-49 was serially passaged in STAT2^−/−^ mice, and virus titers were measured from lung homogenate at day five of infection for various passages by plaque assay. *** *p* < 0.001, **** *p* < 0.0001, One-way ANOVA with Tukey post-hoc test. For (**B**), * *p* < 0.05, ** *p* < 0.01 compared to STAT2^−/−^ P0, One-way ANOVA with Tukey post-hoc test. Data are representative of two independent experiments in A. Data from B are from one experiment, n = 3–5 mice/group or passage.

Some respiratory viruses, including influenza, SARS, and MERS, have been serially passaged in mice to generate mouse-adapted virus strains, which have provided important tools for studying viral and host determinants of disease and tropism [39,40,41,42,43]. We initially sought to generate mouse-adapted HMPV by serially passaging infected lung homogenate directly into a recipient mouse. However, viral titers in lung homogenate of infected C57BL/6 mice were not sufficiently high enough to productively infect a recipient mouse, despite repeated attempts, and serial passage in Rag-2^−/−^ or IFNAR^−/−^ mice was also unsuccessful [30]. However, HMPV replicates to significantly higher titer in IFNAR^−/−^ mice [44], indicating that type 1 interferon signaling restricts HMPV in mice in vivo. We therefore inoculated STAT2^−/−^ mice with HMPV strain TN/94-49 and found that the virus grew to significantly higher titer in STAT2^−/−^ mice compared to STAT1^−/−^ or WT mice (Figure 1A). HMPV could be passaged mouse to mouse at low level detectable by qPCR in STAT1^-/-^ mice, but we chose to pursue STAT2 based on our data showing higher replication in STAT2^−/−^ mice and the fact that the STAT2 protein is less conserved between human and mouse compared to STAT1. We subsequently inoculated STAT2^−/−^ and WT mice with clarified lung homogenate from infected STAT2^−/−^ and WT mice, respectively, and found that HMPV could be serially passaged mouse to mouse in STAT2^−/−^ but not WT mice (Figure 1B). These data show that serial passage of HMPV is restricted by STAT2 in vivo, indicating that this protein and/or the IFN signaling pathway in general is a barrier to murine infection by HMPV.

### 3.2. HMPV Infection Prohibits Nuclear Translocation of STAT1 and STAT2 in Human Cells

**Figure 2:**
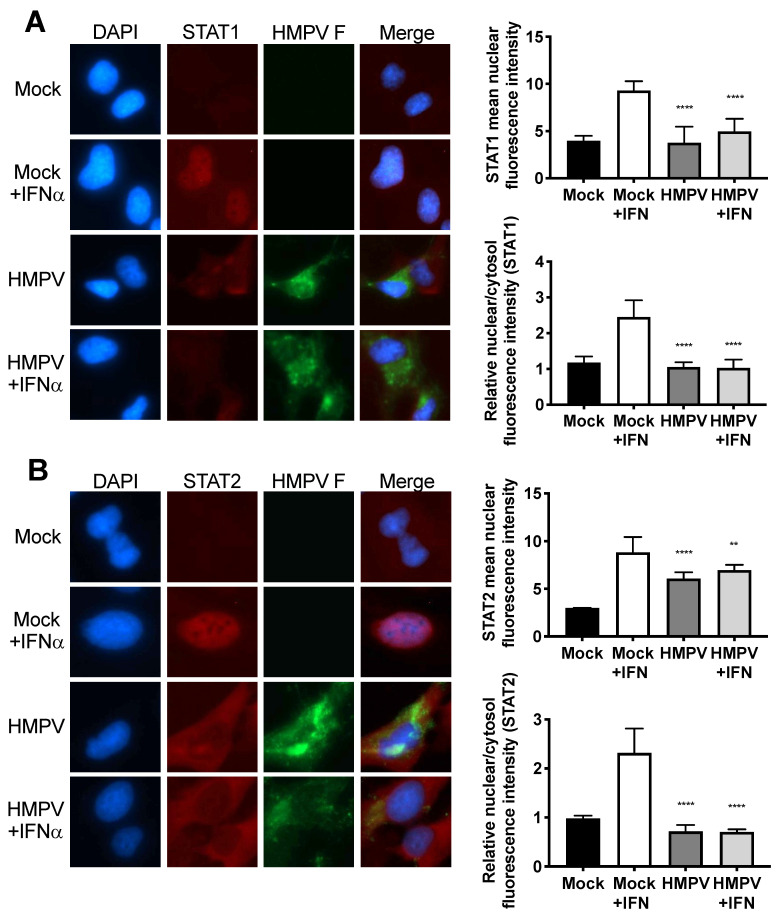
Nuclear localization of STAT1 and STAT2 is inhibited by HMPV. Human BEAS2b cells were infected with HMPV or mock for 20 h, and treated with IFN for 40 min. After IFN treatment, cells were fixed, stained for immunofluorescence, and images were captured at 40× magnification. The fluorescent intensities of the nuclear and cytosolic STAT1 or STAT2 fluorescence signals was quantified. (**A**) HMPV infection inhibits nuclear translocation of STAT1. (**B**) STAT2 nuclear localization is impaired during HMPV infection. Note reduction of STAT1/2 (red) signal in nuclei of HMPV-infected (green) cells. ** *p* < 0.01, **** *p* < 0.0001 compared to Mock + IFN, one-way ANOVA with Tukey post-hoc test. Images are representative of 2 independent experiments. Data in graphs are from two experiments.

Since STAT2 appeared to be important in restricting HMPV infection of mice, we next explored how HMPV infection affects STAT1 and STAT2 in human cells. Previously, it had been shown by us and by others that HMPV can specifically inhibit STAT1 phosphorylation and expression [17,18]. To further understand the effects of HMPV on STAT1 and STAT2, we used a human bronchoepithelial cell line, BEAS2b, to perform imaging studies of STAT1 and STAT2 in the presence or absence of HMPV. After infection with HMPV for 24 h, BEAS2b cells were treated with IFN to induce phosphorylation and nuclear translocation of STAT1 and STAT2. After treatment with IFN, STAT1 and STAT2 translocated to the nucleus in mock-infected cells, whereas nuclear import was inhibited in HMPV-infected cells but not in uninfected cells in the same dish (Figure 2). These data indicate that HMPV infection inhibits nuclear translocation of the STAT1/2 heterodimer in vitro, suggesting that antagonism of STAT1/2 may be a key step to promote HMPV infection.

### 3.3. HMPV Restricts STAT1 and 2 Expression in Primate, but Not Murine, Cells

**Figure 3:**
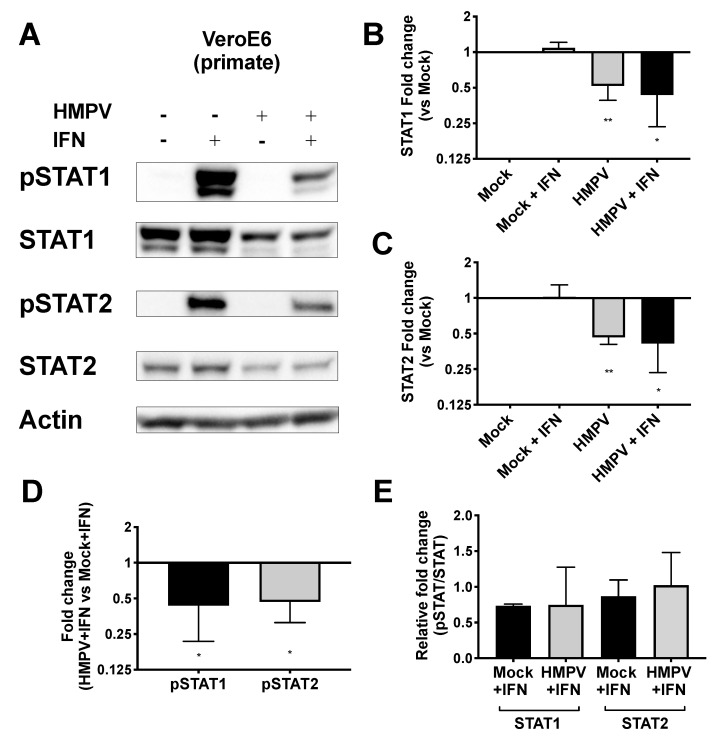
HMPV reduces STAT1 and STAT2 activation in primate cells. (**A**) VeroE6 (primate) cells were infected with HMPV for 24 h, then treated with IFN for 30 min before cells were lysed for Western blotting against total and phosphorylated STAT1 and STAT2. (**B**,**C**) Fold change of total STAT1 (**B**) and STAT2 (**C**) expression normalized to value of mock-infected cells. (**D**) Fold change of pSTAT1 and pSTAT2 compared to mock-infected cells. (**E**) Relative fold change of pSTAT1 and pSTAT2 (fold change of pSTAT1/2 divided by fold change of STAT1/2). * *p* < 0.05, ** *p* < 0.01, one sample *t* test comparison to normalized mock value of 1. Data are representative of (**A**) or combined from (**B**–**E**) four independent experiments.

**Figure 4:**
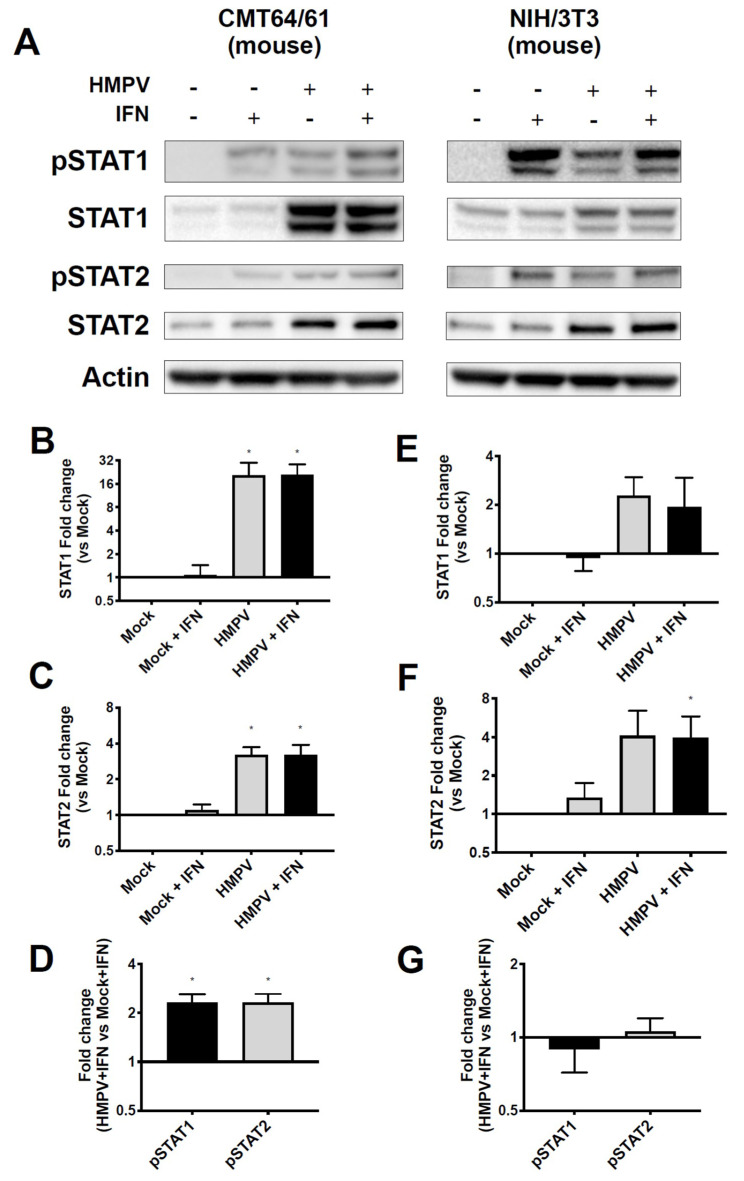
HMPV infection of murine cells leads to upregulation and phosphorylation of STAT1 and STAT2. (**A**) CMT64/61 (murine), left, and NIH3T3 (murine), right, cells were infected with HMPV for 24 h, then treated with IFN for 30 min before cells were lysed for Western blotting against total and phosphorylated STAT1 and STAT2. (**B**,**C**) Fold change of total STAT1 and STAT2 expression compared to mock-infected wells for CMT64/61 cells. (**D**) Fold change of pSTAT1 and pSTAT2 compared to mock-infected wells in CMT64/61 cells. (**E**,**F**) For NIH3T3 cells, fold change of STAT1 and STAT2 compared to mock-infected wells. (**G**) Fold change of pSTAT1 and pSTAT2 compared to mock-infected cells in NIH3T3 cells. * *p* < 0.05, one sample *t* test comparing samples to normalized mock value of 1. Data are representative of (**A**) or combined from four (**B**–**D**) or two (**E**–**G**) independent experiments.

HMPV grew to significantly higher titer in STAT2^−/−^ mice than in WT mice, leading us to hypothesize that HMPV inhibits STAT1 and STAT2 in primate cells but fails to inhibit these in murine cells. To test this hypothesis, we infected both primate and murine cell lines with HMPV and measured expression as well as phosphorylation of STAT1 and STAT2 after IFN treatment. We found that HMPV infection of VeroE6 cells (primate cells that are IFN-responsive but cannot produce endogenous IFN) led to decreased total and phosphorylated STAT1 and STAT2 (Figure 3). The decreased levels of pSTAT1 and pSTAT2 appeared to be driven by the decreased protein abundance of total STAT1 and STAT2 in Vero cells, as quantified by the relative fold change in phosphorylated compared to total STAT proteins. In contrast, when murine cell lines CMT64/61 (C57BL/6 lung adenocarcinoma) and NIH/3T3 (murine fibroblast, deficient in some steps of IFN signaling [45]) were infected with HMPV, we saw increased total and phosphorylated STAT1 and STAT2 (Figure 4). These data indicate that HMPV antagonizes STAT1 and STAT2 in primate cells but fails to achieve this inhibition when introduced to murine cells.

### 3.4. HMPV Inhibition of STAT1 or STAT2 Occurs Independently of the Other

**Figure 5:**
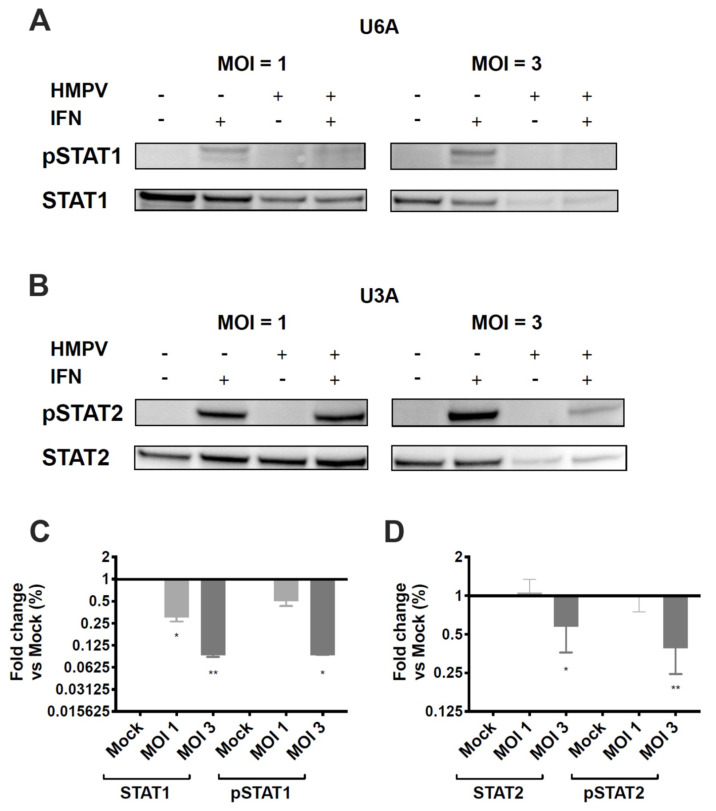
HMPV does not require STAT1 or STAT2 to inhibit expression and phosphorylation of the other. STAT2-deficient U6A (**A**) and STAT1-deficient U3A (**B**) cells were infected with HMPV for 20 h at an MOI of 1 and 3, treated with IFN for 40 min, and lysed for Western blotting. (**A**,**B**) Expression and phosphorylation of STAT1 and STAT2 in U6A and U3A cells. (**C**) Fold change of STAT1/pSTAT1 protein levels in U6A cells. (**D**) Fold change of STAT2/pSTAT2 levels in U3A cells. * *p* < 0.05, ** *p* < 0.01, one sample *t* test to normalized mock value of 1. Data are representative of (**A**,**B**) or combined from (**C**,**D**) two independent experiments.

Studies in hPIV2 and hPIV5 showed that degradation of STAT2 or STAT1, respectively, required the expression of both STAT1 and STAT2 [46]. To understand whether HMPV inhibition of STAT1 and STAT2 is dependent on expression of both proteins, we used U3A and U6A cells, which are specifically deficient in STAT1 and STAT2, respectively [47]. We found that HMPV infection of STAT2-deficient U6A cells led to reduction of STAT1 and pSTAT1 in a dose-dependent manner with increasing MOI (Figure 5A,C). HMPV infection of STAT1-deficient U3A cells also reduced STAT2 and pSTAT2, but only at a MOI of 3 (Figure 5B,D). At MOI = 1 in U3A cells, HMPV did not inhibit STAT2 expression or phosphorylation. These data indicate that HMPV has the capacity to target STAT1 and STAT2 independently of each other; however, antagonism of STAT1 by HMPV appears to be a more efficient process than STAT2 inhibition. 

### 3.5. Expression of hSTAT2 but Not mSTAT2 Promotes STAT1 and STAT2 Inhibition by HMPV

**Figure 6:**
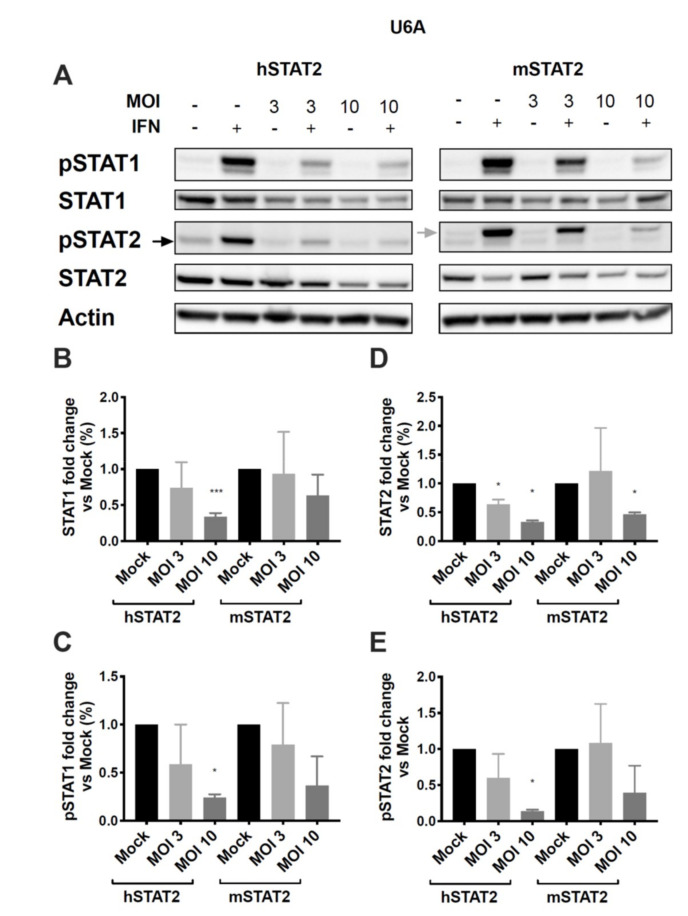
Expression of human STAT2 promotes STAT1 and STAT2 inhibition, while murine STAT2 inhibits STAT degradation. STAT2-deficient U6A cells were transfected with hSTAT2 or mSTAT2, then infected with HMPV. Cells were treated with IFN 16 h after infection for 40 min before cell lysis and protein harvesting for Western blotting. (**A**) STAT1 and STAT2 expression and phosphorylation in U6A cells in the presence of human or murine STAT2. (**B**–**E**) Quantification of band intensity as a measure of fold change in U6A cells for total STAT1 (**B**), pSTAT1 (**C**), total STAT2 (**D**), and pSTAT2 (**E**). * *p* < 0.05, *** *p* < 0.001, one sample *t* test against normalized mock value of 1. Data are representative of (**A**) or combined from (**B**–**E**) two independent experiments.

We next asked whether the species-specific STAT2 inhibition was specifically due to differences in the human and murine forms of STAT2, or whether HMPV fails to inhibit some other step in the innate immune response in murine cells. U6A cells were transfected with either human or murine STAT2 (hSTAT2 and mSTAT2, respectively), infected with HMPV, treated with IFN, and analyzed for STAT1/2 expression and phosphorylation. We found that cells transfected with mSTAT2 were more resistant to inhibition of STAT1 and 2 than those transfected with hSTAT2 (Figure 6). At an MOI of 3, a significant reduction of STAT2 expression was seen in HMPV-infected cells that had been transfected with hSTAT2, but not those transfected with mSTAT2. However, with an increased MOI of 10, reduced STAT2 protein levels were seen regardless of the transfection type (Figure 6D). Interestingly, we found that even though STAT2 was not required for STAT1 inhibition (Figure 5), the presence of mSTAT2 in U6A cells constrained inhibition of STAT1 by HMPV (Figure 6B,C). Overall, these data suggest that species-specific differences in STAT2 limit the inhibition of both STAT proteins by HMPV.

### 3.6. Mice Expressing Human STAT2 Have Greater Disease Severity and Inhibition of ISGs during HMPV Infection

**Figure 7:**
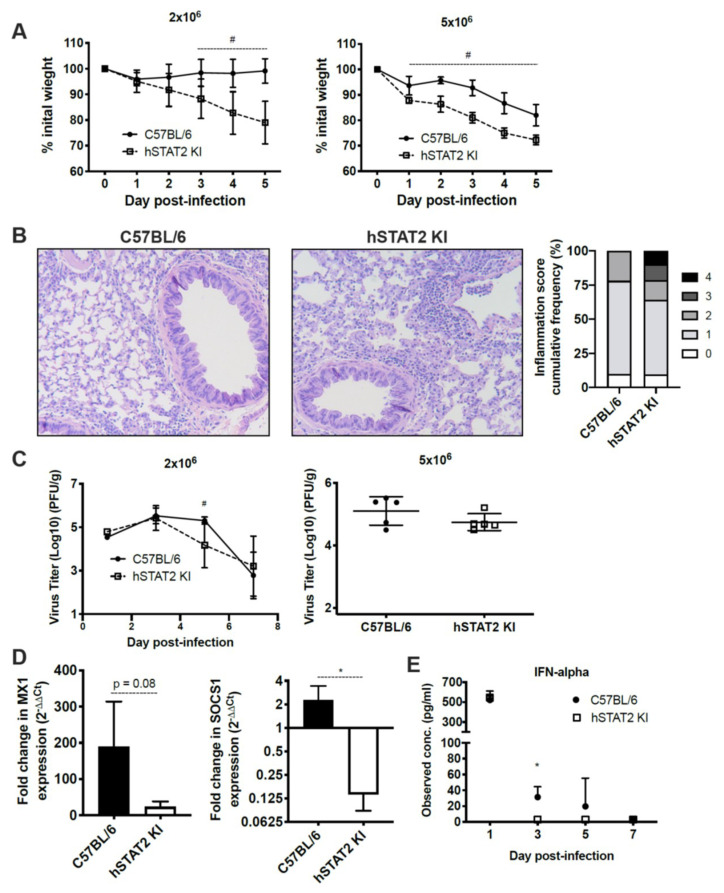
hSTAT2 KI mice have greater disease severity and inhibition of ISGs compared to WT mice after infection by HMPV, despite no increase in virus titer. hSTAT2 KI mice and C57BL/6 mice were intratracheally inoculated with HMPV at a low (2 × 10^6^ pfu/mL) or high (5 × 10^6^ pfu/mL) titer and weighed daily over the course of infection (**A**). (**B**) Lung specimens were taken at day five post-inoculation with HMPV at low titer, stained with H&E, and scored by a pathologist. Histology images were captured at 100×. Histological scoring was calculated by percentage of inflammation per field of view, with scores of 0, 1, 2 (mild/moderate) representing 0, 1–25, and 26–50%, and 3 or 4 (severe) representing 51–75 or 76–100%, respectively. The difference between WT and hSTAT2 KI mice in the frequency of mild/moderate and severe inflammation scores was statistically significant by Fisher’s exact test (*p* = 0.0024). (**C**) Virus titer kinetics in lung homogenate for low inoculum infection (left) or day five virus titers in lungs for high inoculum infection (right). (**D**) qPCR of the ISGs Mx1 and SOCS1 at 24 h post-infection, fold change normalized to mock infection. (**E**) protein concentration of IFNα in lung homogenate at various days post-infection. # *p* < 0.05, 2-way ANOVA; * *p* < 0.05, unpaired *t* test. n = 3–5/group, from two experiments in (**A**–**C**) and from one experiment in (**D**–**E**).

To establish whether HMPV antagonizes the human form of STAT2 compared to murine STAT2 in vivo, we used mice engineered to express human STAT2 in place of murine STAT2 (hSTAT2 KI) [37]. We infected hSTAT2 KI and WT mice with either a low (2 × 10^6^ pfu/mL) or high (5 × 10^6^ pfu/mL) inoculum of HMPV. At both low and high inoculum, hSTAT2 KI mice lost significantly more weight compared to WT controls (Figure 7A). This correlated with a greater degree of lung inflammation (Figure 7B), where hSTAT2 KI mice had a significantly increased frequency of inflammation scores that were categorized as severe (scores of 3 or 4, *p* = 0.0024). The weight loss and increased inflammation was not associated with higher viral titers in infected hSTAT2 KI mice; in contrast, at day five of lower inoculum infection, hSTAT2 KI mice had modestly lower viral burden compared to WT mice, though this was not replicated at higher titer (Figure 7C). These data suggest that, in vivo, STAT2 antagonism worsens disease severity but does not contribute to increased virus replication in mice.

To better understand the ability of HMPV to antagonize interferon signaling in vivo, we quantified levels of IFNα and the ISGs Mx1 and SOCS1 in hSTAT2 KI and WT mice after HMPV infection. IFNα is produced via a feedback amplification loop of ISGs during the type I IFN response [48], while Mx1 and SOCS1 were selected for their roles as known ISGs that have differential functions in the type I IFN response. We found that mRNA expression of Mx1 and SOCS1 were reduced in hSTAT2 KI mice compared to WT mice at 24 h post-HMPV infection (Figure 7D). Additionally, protein level of IFNα declined in hSTAT2 KI mice compared to WT mice during HMPV infection (Figure 7E). Overall, these data indicate that HMPV is indeed able to antagonize type I IFN signaling when mSTAT2 is replaced by hSTAT2 in vivo, though this did not lead to increased virus replication.

### 3.7. HMPV-Infected hSTAT2 KI Mice Demonstrate a Th2-Predominant Cytokine Profile

**Figure 8:**
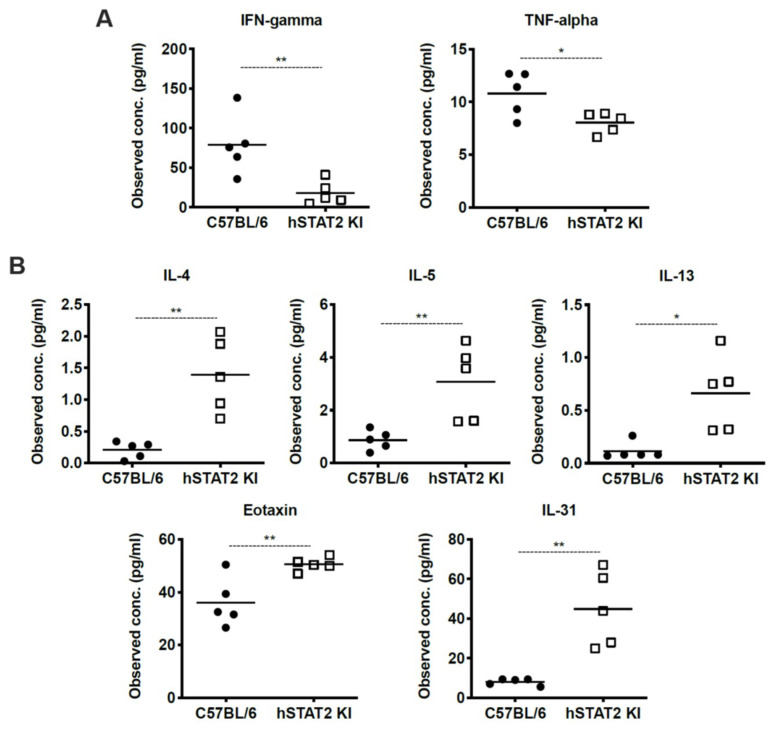
HMPV-infected hSTAT2 KI mice adopt a Th2-skewed cytokine profile. hSTAT2 KI mice and C57BL/6 mice were intratracheally inoculated with HMPV at 5 × 10^6^ pfu/mL. At day five post-infection, lungs were homogenized and cytokines were measured by multiplex cytokine assay. (**A**) Measurement of prototypical Th1 cytokines IFNγ and TNFα in lung homogenates of hSTAT2 KI and C57BL/6 mice. (**B**) Concentration of Th2-related cytokines IL-4, IL-5, IL-13, Eotaxin, and IL-31 in murine lung homogenates. * *p* < 0.05, ** *p* < 0.01, unpaired *t* test. n = 5/group, one experiment.

We hypothesized that the increased weight loss and inflammation despite slightly decreased viral titer in hSTAT2 KI mice was due to aberrant STAT signaling in these mice. To better understand the global consequences of substituting human STAT2 into mice in vivo, we performed multiplex cytokine analysis on lung homogenates of HMPV-infected hSTAT2 KI and WT mice at day five after high-dose inoculation, as day five represents a time point that bridges innate and adaptive immunity. Overall, hSTAT2 KI mice had reduced expression of the characteristic Th1 cytokines IFNγ and TNFα (Figure 8A) and adopted a Th2-skewed cytokine profile demonstrated by increased concentrations of IL-4, IL-5, IL-13, eotaxin, and IL-31 (Figure 8B). Altogether, our data suggest that the substitution of hSTAT2 for murine STAT2 leads to skewing of both the innate and the adaptive immune response to HMPV. 

## 4. Discussion

Animal models are essential tools for studying infections by human viruses. The differences in host responses between humans and animals, both in vitro and in vivo, help reveal important mechanisms of how viruses interact with the immune system. We showed that HMPV can be serially passaged in STAT2-deficient mice, whereas serial passage in WT mice was not possible, suggesting a role for STAT2 in mediating in vivo restriction. HMPV inhibited expression and nuclear localization of STAT1 and STAT2 in primate airway epithelial cells, while the virus failed to inhibit either STAT in murine cells. STAT inhibition occurred in a hSTAT2-dependent manner, as transfection of mSTAT2 into human cells reduced STAT1 and STAT2 antagonism. Furthermore, the knock-in of hSTAT2 into mice enabled HMPV to inhibit type I IFN signaling and alter the cytokine profile of infected mice. Collectively, these data suggest that STAT2 in mice represents a significant barrier to murine infection by HMPV.

We observed that a relatively high MOI of HMPV (three or more) was needed to inhibit STAT2, whereas inhibition of STAT1 occurred at a MOI of one. These effects at different MOIs suggest that STAT1 inhibition by HMPV is more efficient than STAT2 inhibition. HMPV proteins could have a higher affinity for STAT1 compared to STAT2, or perhaps STAT2 inhibition is achieved with a viral protein that is transcribed at a lower abundance. Further research to elucidate which HMPV protein is responsible for antagonizing STAT2 will increase our understanding of how HMPV inhibits different steps of the innate immune response. 

A limitation of the in vitro component of this work is that cell lines may have inherent differences in the strength of their innate immune signaling, in addition to the differences in the efficiency of infection and transfection between cell lines. Previous work in our lab showed that autocrine and paracrine effects of IFN signaling on neighboring uninfected cells could confound the effects of HMPV infection on STAT1 expression and phosphorylation [18]. Here, we attempted to control for this effect in primate cells by using Vero E6 cells, which cannot produce endogenous IFN. However, no equivalent murine cell line currently exists, though we opted to use the murine NIH/3T3 cell line for experiments in Figure 4, as this line has some known impairments in endogenous IFN signaling [45,49]. 

Several prior studies have described species-specific differences in STAT2 inhibition by the flaviviruses Dengue, Zika, and Yellow Fever viruses [50,51,52]. Consequently, hSTAT2 KI mice infected with mouse-adapted Zika virus exhibited increased viral replication, broader tissue spread, and enhanced transplacental spread [37]. In contrast, we found that despite the clear preference for inhibition of hSTAT2 over mSTAT2 in vitro, HMPV-infected hSTAT2 KI mice had similar virus replication in the lung compared to WT mice. Nevertheless, the presence of hSTAT2 resulted in reduced expression of IFNα and Mx1 in these mice, consistent with inhibition of STAT1/2 signaling, although not to a sufficient level to result in a significant increase in viral replication in lung. Interestingly, hSTAT2 KI mice had worse disease than WT mice when infected with HMPV, as measured by weight loss and lung inflammation. 

This phenotype was associated with an altered cytokine profile that favored Th2 cytokines over Th1. Since IFNα was significantly reduced in hSTAT2 KI compared to WT mice, it is likely that either direct or indirect inhibition of STAT2 by HMPV drove the Th2 phenotype in this study. IFNs and IL-4 have opposing roles in the immune response, with IFNs promoting an antiviral Th1 response and IL-4 driving a Th2 response characterized by asthma and allergy. This response is driven in part by IFN-mediated inhibition of IL-4 via increased SOCS1 expression [53]; our data here are consistent with this previous work, as we saw increased IL-4 and other Th2 cytokines while IFNs and SOCS1 were reduced (Figure 7 and Figure 8). Due to the dramatic skewing of the Th1/Th2 axis in the presence of hSTAT2, we hypothesize that antagonism of STAT2 by HMPV in human infection may contribute to the association of HMPV and asthma in susceptible individuals [54,55,56]. In addition, STAT2 has immunoregulatory functions that may have been disrupted in the hSTAT2 KI mice [57,58,59].

Overall, these data indicate that HMPV targets expression of both STAT1 and STAT2 in a host-specific manner. Future studies to determine the specific HMPV protein(s) and molecular interactions involved in STAT inhibition will reveal mechanisms of productive infection and pathogenesis in humans. While mouse models of human diseases have strengths and limitations, these data highlight how the innate immune response to HMPV in mice may be profoundly different than it is during natural infection of humans, an important consideration for the development of future therapeutics and vaccines.
